# Exploration of health care providers' knowledge-based care and support given to family members and caregivers of people living with epilepsy

**DOI:** 10.3389/fpsyg.2024.1396874

**Published:** 2024-07-23

**Authors:** Ofhani Prudance Musekwa, Lufuno Makhado, Angelina Maphula

**Affiliations:** ^1^Department of Psychology, Faculty of Health Sciences, University of Venda, Thohoyandou, Limpopo, South Africa; ^2^Office of the Dean for Research, Faculty of Health Sciences, University of Venda, Thohoyandou, Limpopo, South Africa

**Keywords:** epilepsy, epilepsy support, caregiver, care, family member, health care provider, support

## Abstract

**Introduction:**

In South Africa, approximately half a million individuals live with epilepsy. This means that half a million families and caregivers are impacted by epilepsy, with a limited number of healthcare providers responsible for treating people living with the condition, as well as their families and caregivers.

**Methods:**

This study explored the knowledge-based care and support healthcare providers give families and caregivers. Fifteen participants were purposefully selected from Limpopo and Mpumalanga provinces to participate in the study. Data were collected via an open-ended interview guide divided into two sections: Section A comprised sociodemographic questions, and Section B had questions on epilepsy care and support. Four of the participants were male, and 11 were female, seven were community health workers, five were nurse practitioners, and three were auxiliary nurses. Seven had a grade 12 qualification or lower, and only six had a degree. The data collected was analyzed using thematic analysis, coded by the researcher and co-coded by an independent expert. Two themes emerged from the raw data: epilepsy knowledge and epilepsy support and counselling. From these two themes, three subthemes were identified: psychosocial impact of epilepsy, epilepsy-related training, and counselling and support.

**Results:**

The study revealed a gap in professional capacity building and highlighted the need for intentional knowledge sharing and equipping of healthcare providers.

**Discussion:**

The findings suggest that equipping community health workers, in particular, may be a better and more efficient way to increase the quality of life for families and caregivers and people living with epilepsy in South Africa.

## 1 Introduction

Over fifty million people worldwide are affected by epilepsy (World Health Organization, 2019), which in turn impacts millions of families who must care for a loved one with epilepsy. In South Africa, more than half a million individuals live with epilepsy, suggesting that perhaps two to three times that number of individuals are impacted by this medical condition (Epilepsy South Africa, 2023). It was estimated that by age 20, ~75% of people living with epilepsy (PLWE) will experience their first seizure (Epilepsy South Africa, 2023). In sub-Saharan Africa, the prevalence of active epilepsy was projected to increase within the age range of 20–25 years (Paul et al., 2012), pointing to the need for attentive care from a young age. In Eastern Africa, the highest prevalence of epilepsy was reported among individuals aged 15–29 years (Stelzle et al., 2022). The International League Against Epilepsy (ILAE) reported that incidences of epilepsy are age-dependent (commonly found in children younger than 5 years and adults older than 65 years; Zuberi et al., 2022). More specifically, in Nova Scotia and Minnesota, the onset of epilepsy was found to be much higher within the 1st year of life (Zuberi et al., 2022). This also puts into perspective the longevity of the impact of epilepsy on the lives of those affected, either directly or indirectly (Berg et al., 2019; Pokharel et al., 2020).

Although trained professionals were available to care for and monitor PLWE, family members tended to spend the most time caring for their loved ones. A nursing handbook on patient safety and quality reports that family members spend over 40 h a week on nursing care (Hughes, 2008). This may be more applicable to the family's primary caregiver, as they would assume more care responsibilities. Consequently, the time spent paying attention to the patient may hinder the healthy development of other siblings, especially if the primary caregiver is a parent or an older sibling. This may give rise to feelings of caregiver guilt and an increased burden on the family (International League Against Epilepsy, 2019).

Family members and caregivers often feel ill-equipped to provide adequate epilepsy care because they lack proper guidance and attention from healthcare professionals (Hughes, 2008; England et al., 2012; Musekwa et al., 2023). Moreover, nurses have a limited perspective of family members' duty toward patient care (Hughes, 2008), which may result in a lack of health providers' involvement in family members' training and support. The lack of focus on caregivers is considered a healthcare gap, posing potential risks and harm to patients (Hughes, 2008). Thus, knowledge-based initiatives should prioritize educating healthcare professionals (HPs) and community health workers (CHWs) who are expected to transfer their knowledge and caregiving expertise to family members and caregivers.

Inadequate knowledge of epilepsy among healthcare providers and community health workers (CHWs) has significant implications for the care and support provided to individuals with epilepsy. Misdiagnosis and delayed treatment can occur when understanding of the condition is limited (Auvin et al., 2018; Blazejewski et al., 2018). This can result in delayed initiation of appropriate treatment, potentially exacerbating the frequency and severity of seizures and increasing the risk of complications. In addition, ineffective treatment management may occur when healthcare providers lack the knowledge to manage epilepsy treatment effectively (Ghaziuddin et al., 2004; Kerr et al., 2011; World Health Organization, 2015; Arinda et al., 2023). They may struggle with medication regimens, drug interactions, and recognizing the importance of treatment adherence, which can compromise the effectiveness of treatment and hinder seizure control.

Insufficient understanding of epilepsy may prevent healthcare providers from providing appropriate guidance and emotional support (Nyblade et al., 2009; Snape et al., 2009; Engel and Rempel, 2016; Adugna et al., 2020). Consequently, PLWE and their caregivers may experience increased stress, anxiety, and psychological burden. Additionally, limited knowledge can contribute to stigma and social exclusion (Tran et al., 2007; Hirfanoglu et al., 2009; Braga et al., 2020; Ali Arazeem et al., 2022). Inaccurate beliefs and misconceptions about epilepsy may be perpetuated, further isolating PLWE from society.

Inadequate knowledge can hinder access to resources and services for individuals with epilepsy. Healthcare providers and CHWs who are not well-informed about epilepsy may not be aware of available educational materials, support groups, specialized clinics, and referral options (Elliott and Shneker, 2008; Keikelame and Swartz, 2013). This can limit the comprehensive care and support that individuals with epilepsy receive, especially in rural areas with limited access to information and resources.

To ensure appropriate care for PLWE, addressing the inadequate knowledge of epilepsy among healthcare providers and community health workers is crucial for improving their overall care and quality of life. Comprehensive education and training programmes should be implemented to enhance their understanding of epilepsy and its management (Bishop et al., 2021). By equipping healthcare providers and community health workers with accurate knowledge, PLWE can receive better care and support, improve outcomes, and reduce patient and family burdens. It is, therefore, imperative to explore the epilepsy knowledge and understanding of healthcare providers. For this reason, the researchers conducted this study to explore the knowledge and support given by healthcare providers regarding epilepsy.

## 2 Materials and methods

This article is based on the second phase of an explanatory sequential study. The first phase was a quantitative non-experimental survey of 102 healthcare provider respondents who participated in an instrument piloted on HCPs in Limpopo Province. This phase showed that HCPs had somewhat sufficient knowledge of epilepsy with persisting negative attitudes, concluding that there is a misalignment between HCPs' knowledge and understanding of epilepsy. In addition, HCPs showed that they are not receiving regular training on epilepsy care. In this current phase, the researchers adopted a phenomenological qualitative case study method to answer the question: Are healthcare providers sufficiently capacitated to provide quality epilepsy care and support to family members and caregivers of PLWE? Based on the outcome of the first phase, all health providers were eligible for participation. However, 15 participants were included to participate in the study using purposive sampling. Data were collected at Jerusalem Clinic in Mpumalanga and Bochum Clinic in Limpopo province, where nurse practitioners (NPs), auxiliary nurses (ANs), and CHWs worked. These clinics are rural-based and strategically placed to serve local and surrounding communities. However, the infrastructure of both clinics was somewhat poor. In addition, the clinic had awareness posters on various illnesses like HIV and cancer but none on epilepsy.

All the participants were granted permission to participate as they knew about the study, and rapport had already been established. However, the researcher telephonically contacted participants who expressed interest and showed participation availability for participation confirmation. The researcher described the nature of the second phase, its expectations, and how it will be run before commencement. After the participants had agreed to participate, the researcher gave them a meeting date and time.

Before starting with the face-to-face semi-structured individual interviews, each participant was required to sign a consent form before commencing with interviews. Data collection took 30–40 min with each individual participant within the confines of a private office. The researcher used an audio recorder and took notes during the interviews. The data collection tool comprised two sections: Section A consisted of sociodemographic questions, and Section B asked questions on epilepsy knowledge (source of knowledge or information and impact), epilepsy training (training received and offered to family members and caregivers), and counselling offered to family members and caregivers. In Mpumalanga, saturation was reached by participant five and participant four in Limpopo. In both provinces, three more participants were interviewed to ensure that there would be no new information that is generated post-saturation The researcher transcribed and analyzed the collected raw data using thematic analysis as described by Clarke and Braun (2017). An independent co-coder simultaneously analyzed the data along with the researchers to ensure trustworthiness. The researchers and co-coder agreed on the data codes and themes; two main themes and three subthemes emerged from this process.

## 3 Findings

### 3.1 Socio-demographic data

As shown in Table 1, the study involved 15 participants, comprising three ANs, five NPs, and seven CHWs. These participants were recruited from two provinces in South Africa, namely Limpopo and Mpumalanga. Among the participants, four were male and nine were female. In terms of education, seven individuals had completed grade 12 or lower, six had obtained a degree, and two held post-graduate degrees. When asked about their prior experience interacting with family members or caregivers of individuals with epilepsy, 13 participants acknowledged having had such conversations.

**Table 1:** Socio-demographic data.

**Items**	**Categories**	**Participants (*n*)**
Province	Limpopo	7
	Mpumalanga	8
Gender	Male	4
	Female	11
Profession	Auxiliary nurse (AN)	3
	Nurse practitioner (NP)	5
	Community Health Worker (CHW)	7
Level of education	Grade 12/lower	7
	Degree	6
	Postgraduate studies	2
Years of experience	Less than 10 years	3
	Between 10–15 years	1
	From 15–20 years	6
	Above 20 years	5
Have you ever spoken to a family member/caregiver to a PLWE?	Yes	13
	No	2

### 3.2 Themes

**Figure 1:**
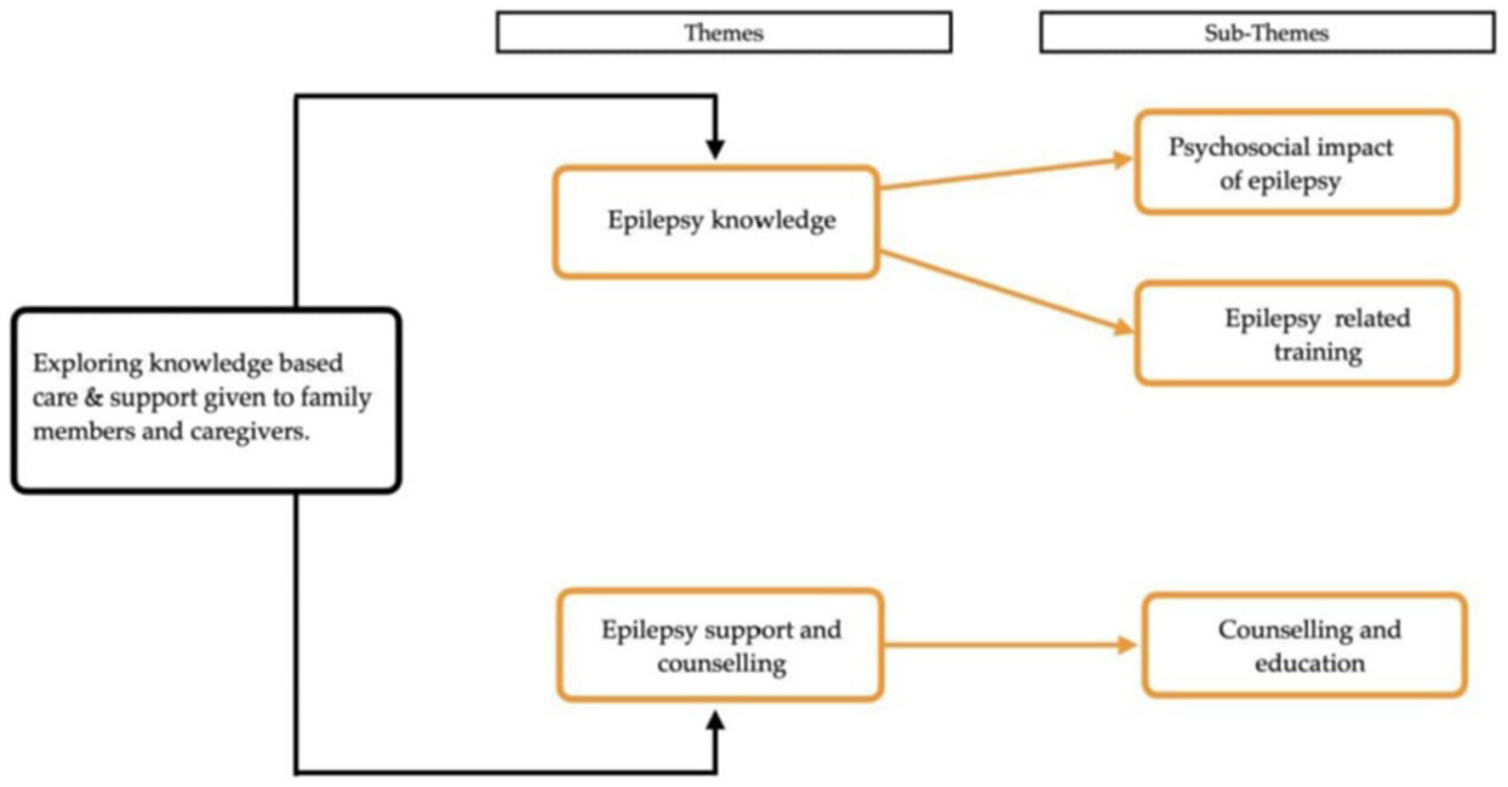
Study themes and sub-themes.

Two themes and three subthemes emerged from this study, as illustrated in Figure 1. The themes included epilepsy knowledge and epilepsy support and counselling. From the theme of epilepsy knowledge, two subthemes emerged: the psychosocial impact of epilepsy and epilepsy-related training. From the second theme on epilepsy support and counselling, the subtheme that emerged was epilepsy counselling and education.

#### 3.2.1 Theme 1: epilepsy knowledge

Participants in this study knew about epilepsy and understood that it is a health problem, especially for the patient and the family. Many (*n* = 9) emphasized that continued adherence to medication would not have a detrimental effect on the lives of PLWE. Participants also demonstrated, through their comments, their awareness of issues of stigmatization toward PLWE, acknowledging the psychological and social complexities associated with the disorder:

“*…they are so many people who are affected with epilepsy, such as their family and friends, and still there is stigma uhm around it.”* (Participant 5, AN)

“*Those who are under proper treatment can have the condition under control. If one does not follow a proper procedure of taking treatments, the condition becomes a problem.”* (Participant 11, NP)

##### 3.2.1.1 Subtheme 1: psychosocial impact of epilepsy

This current study revealed that many participants (*n* = 11) believed that living with epilepsy had a psychosocial impact. However, most appeared to have more knowledge of the social rather than the psychological aspects of the condition. Participants confirmed that epilepsy results in social challenges such as social isolation, dropping out of school, and a lack of social skills. Some participants also reported self-negligence, family burden, and shame. They claimed that patients suffered psychologically from short-term memory loss, difficulties with anger regulation, a short attention span, anxiety, and generally appeared to be mentally disturbed:

“*Most people have a problem at school, and they need to be monitored. They cannot be home alone, and psychologically, if you have epilepsy, you tend to forget everything.”* (Participant 1, NP)

“*… when you look at them, you think they are mentally disturbed. Some do not even pay attention to you, and they have anger…”* (Participant 10, CHW)

“*When the moon glows or appears at night, they become anxious…”* (Participant 7, NP)

“*Others, they are always sitting down. You would barely see them walking around.”* (Participant 12, AN)

“*… they need to be cared for at every time. It's a problem to the family members as they cannot go out to other things or attend other important aspects and leave the patient home alone.”* (Participant 4, CHW)

“*The person living with epilepsy does not recall or remember what happened when they collapsed… others neglect themselves...”* (Participant 2, NP)

##### 3.2.1.2 Sub-theme 2: epilepsy-related training

This study revealed that CHW from Mpumalanga received training on epilepsy. However, those from Limpopo only received formal training on epilepsy at the inception of the study when the research team engaged with the participants. Moreover, this study showed that some (*n* = 5) participants who are NPs stated that they only learned about epilepsy at school and did not receive any formal training after graduating. It was further revealed that, to some extent, CHWs receive more training about epilepsy than NPs.

All CHWs agreed to have received some form of epilepsy training. However, most were uncertain about the frequency of the training they had received, and those who could recall, reported being trained only once (*n* = 4). Almost all participants reported that the training that did take place only addressed epileptic seizure first aid. One participant added that they were taught about the impact of epilepsy on an individual's life, and another stated that they educated themselves:

“*But because now we are learned, we know that putting something in the mouth is prohibited to do so. We were taught that you help the individual sleep on one side.” “We get training every now and then in the facility.”* (Participant 9, CHW)

“*They tell us that we should teach others about this illness and people should take it seriously and not hide the person who might have it.”* (Participant 5, AN)

“*I've only done the training while I was still at school [Tertiary Education]…there are no workshops for that or anything like that.”* (Participant 14, NP)

“*I last heard or read about epilepsy while I was at school doing my 3rd year and I'm using that knowledge up to today to work with epileptic patients. We hardly have any workshops on epilepsy. Sometimes I have to use the internet to be updated.”* (Participant 7, NP)

“*I received it once in 2019 for 3–4 days when you came…”* (Participant 10, CHW)

#### 3.2.2 Theme 2: epilepsy support and counselling

Participants in this study mostly offered support to the family members and carers of PLWE, especially CHWs, who visited the families of these patients regularly. They also invested more time in the family members and primary caregiver than NPs, who seemed detached from direct support to patients and their family members:

“*I counsel everyone in the family when I make home visits for health talks 4 times a month.”* (Participant 6, CHW)

“*The clinic is so busy. We have so many different patients, I cannot just give information to everyone who does not need it. So, whenever they come to get medication, if they have a question on anything, we give them health education and answer them, but if not, we move on to the next patient.”* (Participant 15, CHW)

##### 3.2.2.1 Subtheme: counselling and education

All but three participants stated that they offered primary caregiver training to patient caregivers and family members. They maintained that although no set material prescribes what should be taught, the training and education content includes first aid and treatment adherence. This training is usually given to the primary caregiver. In addition, the participants who acknowledged not offering training also mentioned that they supplemented it with counselling sessions. Some mentioned that, while counselling, they spoke to caregivers about medication and encouraged them to ensure patients kept taking their medication. Others stated that during their visits, they checked on how caregivers were coping with caring for the patient and had general conversations about epilepsy:

“*I train them on how to handle the patient if he experiences a seizure and how to provide aid and care for the patient.”* (Participant 3, CHW)

“*I do offer training based on my skills and knowledge to the family of the person living with epilepsy, especially the caregiver.”* (Participant 10, CHW)

“*So there's no information that is readily available to give to the family members…”* (Participant 2, NP)

“*Mostly, the people living with epilepsy, I think we don't care much about them. We can say that because, with epileptic patients, we don't have the training and even something to teach, unlike HIV patients.”* (Participant 11, NP)

“*There is a boy who lives with their grandmother, and we teach her [grandmother] that they should frequently go to the clinic to get pills for the boy and make sure he drinks them at the right time.”* (Participant 5, AN)

## 4 Discussion

This study explored knowledge-based epilepsy care and support for family members and caregivers in rural Limpopo and Mpumalanga. It was found that similar to a study conducted by Assadeck et al. (2020), NPs and other non-professionals (CHWs in the case of this study) showed different variations of impact on care and epilepsy support. In both instances, non-professional nurses were more involved in epilepsy care and support. In this study, CHWs showed more engagement with the patients' families. Although CHWs frequently visited the families, this study revealed that the nature of their visits was mainly concerned with issues of epilepsy first aid and adherence to treatment and was aimed at engaging the primary caregiver. However, some studies suggest that the presence of specialized community nurses bridges the gap between the illiteracy of CHWs and the limited involvement of NPs, while offering the best of both worlds (Kerr et al., 2011; Higgins et al., 2019; Hutchinson et al., 2022). This would be of great benefit to PLWE. However, considering the global prevalence of epilepsy and the economic strain on low- to middle-income countries, epilepsy education for healthcare providers (intentionally repetitive training and information sharing) may be the most viable option, especially in rural locations.

Interestingly, NPs and ANs, who serve as first-contact sources of knowledge (during home visits or at the health facilities), displayed an inadequate knowledge of epilepsy and often employed inappropriate practices toward PLWE, further questioning HCPs competence in providing health (Suresh et al., 2017; Buddhiraja et al., 2020; Asadi-Pooya et al., 2022; Musekwa et al., 2023). Although previous research (Wagner et al., 2020) has found that traditional preferences were a contributing factor to the lack of adherence to treatment (Wagner et al., 2020; Nemathaga et al., 2023), this study suggests that inadequate training (especially in information delivery) could also be a reason behind the reported lack of adherence, despite encouragement from CHWs. Moreover, post-diagnosis, knowledge sharing with the rest of the family and the patient is often neglected.

These factors all center around effective knowledge acquisition and dissemination. Insufficient knowledge or the ability to share essential information adequately may ultimately diminish the quality of life for PLWE and their families (Kiwanuka and Anyango Olyet, 2018; Musekwa et al., 2023; Nemathaga et al., 2023). While it may appear to be the case, it is worth noting that CHWs can provide support in the form of home visits, provided they receive proper training. In addition, as a form of epilepsy support method, family members and caregivers may benefit from monitored support groups that have proved successful (Tran et al., 2007; Chung et al., 2012; Elafros et al., 2013; Kiwanuka and Anyango Olyet, 2018; Miller et al., 2020; Muche et al., 2020; Evett et al., 2021).

The lack of and need for intentional support, training, and education were evident in the study's findings. Little to no education is available to facilitate epilepsy care and support, as Yu et al. (2022) also suggested in their study. The findings of this current study indicate that more knowledge and training should be provided among CHWs. Some even stated that they had only been trained once, and NPs and ANs stated that they had never received training after graduating.

## 5 Limitations and recommendations

The data collection tool may have been a limitation—inconsistencies were noted with the quantitative findings, as healthcare workers stated that they offered counselling but could not adequately describe the content of the counselling given. This may be because participants did not understand what counselling entails. Researcher bias may have impacted the study because of the researchers' professional counselling knowledge.

Because of the qualitative elements of the study, the researchers were unable to measure the level of epilepsy care and the comparative impact between CHWs and NPs. However, this presents an opportunity for future study—comparing the impact of CHWs and NPs in epilepsy patient and family member care and support. Another potential avenue for future investigation is determining the need for epilepsy-specific health practitioners in rural South Africa.

## 6 Conclusion

This study underscored the significance of knowledge-based epilepsy care and support from health providers (practitioners and CHWs). It also highlighted the gap and the critical need for professional capacity building for these individuals. In addition, the study emphasized that intentional and continuous education is essential to provide the best care to PLWE through collaboration and knowledge transfer to family members and caregivers. Furthermore, this study showed that developing a support structure for family members and caregivers is crucial to enhancing their quality of life. Health institutions/departments should also prioritize basic counselling training and provide regular information to all healthcare providers regarding epilepsy. Additionally, a standardized information template for all health practitioners will ensure uniformity, consistency of information shared, and rigor. Epilepsy training, mentorship, and capacity building for NPs and CHWs should be prioritized and set apart as necessary. Given the potential for CHWs to become the cornerstone of epilepsy care, health systems should focus on equipping them with care and support, considering their proximity to patients and their families.

## Data availability statement

The datasets presented in this article are not readily available to protect participant anonymity. Requests to access the datasets should be directed to OM, ofhanimusekwa@gmail.com.

## Ethics statement

The studies involving humans were approved by the Human and Clinical Trial Research Ethics Committee of the University of Venda. The studies were conducted in accordance with the local legislation and institutional requirements. The participants provided their written informed consent to participate in this study.

## Author contributions

OM: Conceptualization, Data curation, Investigation, Methodology, Writing – original draft, Writing – review & editing. LM: Funding acquisition, Supervision, Writing – review & editing. AM: Supervision, Writing – review & editing.
